# Which family members use the best nets? An analysis of the condition of mosquito nets and their distribution within households in Tanzania

**DOI:** 10.1186/1475-2875-9-211

**Published:** 2010-07-22

**Authors:** Angela Tsuang, Jo Lines, Kara Hanson

**Affiliations:** 1 London School of Hygiene and Tropical Medicine, Keppel Street, London, WC1E 7HT, UK

## Abstract

**Background:**

Household ownership of insecticide-treated mosquito nets (ITNs) is increasing, and coverage targets have been revised to address universal coverage with ITNs. However, many households do not have enough nets to cover everyone, and the nets available vary in physical condition and insecticide treatment status. Since 2004, the Government of Tanzania has been implementing the Tanzania National Voucher Scheme (TNVS), which distributes vouchers for ITNs through antenatal clinics to target pregnant women and their infants. This analysis aimed to determine the following: (1) coverage patterns of bed nets *within *households according to physical condition and treatment status; (2) who might be at risk if mosquitoes were diverted from occupants of untreated nets to those not using nets? (3) the degree to which those at highest risk of malaria use the most protective nets.

**Methods:**

Data from the 2006 TNVS household survey were analysed to assess within-household distribution of net use. The associations between net characteristics and net user were also evaluated. Multivariate analysis was applied to the relationship between the number of holes per net and user characteristics while adjusting for confounders.

**Results:**

In households with a net:person ratio better than 1:4 (one net for every four household members), more than 80% of the people in such households reported using a net the previous night. ITNs were most likely to be used by infants, young children (1-4 y), and women of childbearing age; they were least likely to be used by older women (≥50 y), older children (5-14 y), and adult men. The nets used by infants and women of childbearing age were in better-than-average physical condition; the nets used by older women and older children were in worse-than-average condition; while young children and adult men used nets in intermediate (average) condition. When adjusted for confounders, the nets used by young and older children had more holes than nets used by infants.

**Conclusions:**

Infants and other vulnerable groups were most likely to sleep under the most protective nets. Nevertheless, more communication efforts are needed to increase use of intact ITNs within households for children. Further research is necessary to fully understand motivations influencing within-household net distribution.

## Background

Malaria poses a serious threat to pregnant women, infants, and children [1,2], and these groups are at particular risk in high transmission settings. Insecticide-treated bed nets (ITN) have been proven to be highly effective against mosquito bites and therefore preventing the transmission of malaria [3]. Substantial progress has been made in increasing ITN use by those who are at greatest risk [4], and more recently the UN Secretary-General has called for "universal coverage, by the end of 2010 . . . to all people at risk of malaria, especially women and children in Africa" [5,6]. In order to achieve universal coverage in places with increasing but still partial coverage, the plans of ITN programme managers may seek to take account of the pre-existing coverage, i.e. the nets that are already in domestic use.

Because nets are supplied by local NGOs and commercial distribution systems as well as national programmes, the population of nets in a community at any one time is likely to be composed of nets of a wide range of ages, sources, and qualities. We focus here on the processes that are expected to reduce the effectiveness of nets with age. First, in the case of ITNs, there is a gradual loss of insecticide over time, reducing their protective effect. Second, as a result of wear and tear, nets will accumulate holes. Although recent studies have started to focus on the rate at which holes appear, almost nothing is known about the factors that might influence this process. Overall, the available evidence suggests the existence of wide variation, between households and between countries, in the rate at which protection is lost through wear and tear, and presumably therefore in the epidemiological protection given by the net [7].

People also differ in their vulnerability to the effects of malaria. Pregnant women, particularly primagravidae, are recognised to be at high risk with malaria in pregnancy associated with both more serious illness among the women themselves and with low birth weight [8,9]. Mortality due to malaria in Africa is highly concentrated in younger children. For example, one demographic surveillance study in Burkina Faso recorded 732 malaria-attributable deaths in children over the period 1999-2003; of these deaths, 36% occurred in infants less than one year old, 56% in children aged one to four years, and 8% in children aged five to fourteen years [10]. The situation is very similar elsewhere in Africa. Compiling data from seven demographic surveillance sites in Africa, Abdullah et al (2007) examined the age-distribution of deaths due to malaria in children under fifteen years, and found that the median age at death of these children was very young, ranging from 1.01 to 1.65 years across the various sites [11]. Given these differences in risk, it is therefore important to know whether family preferences in net usage lead to an association between the condition of the net and the vulnerability of the user.

Since many existing nets are untreated (or previously treated, but with insecticide which has worn off), an additional concern is exposure to the so-called "diversion effect," whereby mosquitoes can be diverted within a room from one person using an untreated net to an unprotected person. However, if the net is treated, there is evidence that the "diversion effect" does not occur because the insecticide offers some protection to non-net-users sleeping nearby [12]. Thus, determining preferences for the use of treated and untreated nets among family members is of value.

In effect, these are questions about within-family equity. Previous studies have investigated the equity of net coverage in terms of differences in household ownership or individual use, by linking these to socioeconomic status [13-15]. However, the equity of net distribution *within *households in terms of net condition and treatment status has not been examined. More specifically, these questions concern the concept of vertical equity, which exists if those with greater health needs are treated preferentially [16,17]. The objective of this paper is to evaluate the relationship between the physical condition of nets and individual user characteristics, in terms of both the number of holes and insecticide treatment status, in order to assess the vertical equity of within-household net distribution in net-owning households. We do this by restricting some of the analysis to households which have at least one infant or child under five years old.

### Study setting

The data used for this analysis come from the 2006 round of the household survey undertaken as part of the monitoring and evaluation of the Tanzania National Voucher Scheme (TNVS). Malaria is prevalent in all districts of Tanzania. The predominant parasite is *Plasmodium falciparum *and the main vectors are *Anopheles gambiae *and *Anopheles funestus*, with peak malaria transmission occurring during the rainy seasons between November and May.

Commencing in October 2004, the National Malaria Control Programme (NMCP) of the Government of Tanzania implemented the TNVS (also known as *Hati Punguzo*) in all 21 regions of the mainland to distribute ITNs through antenatal clinics (ANC), targeting pregnant women and their newborn infants. The roll-out of this programme was completed nationwide by May 2006. From 2005 to 2008, a research team from the London School of Hygiene & Tropical Medicine and the Ifakara Health Institute was contracted to evaluate the TNVS [18,19].

Through the TNVS, discount vouchers are distributed to pregnant women during their first antenatal care visit. The voucher provides a subsidy towards the cost of an ITN purchased from a local retailer. Therefore, these vouchers increase the likelihood that an ITN will be used by the pregnant woman and subsequently her newborn infant [20]. The aim of the TNVS to target pregnant women and infants, because of their biological vulnerability to malaria, is an example of vertical equity. Because pregnant women, infants, and children under age five are at greatest risk for severe malaria, there would be vertical equity within households if these groups were most likely to sleep under intact ITNs.

## Methods

The data come from a nationally representative, cross-sectional household survey conducted in 2006 from approximately 6300 households across 21 districts of Tanzania. Within each randomly-selected district, 10 clusters of 30 households were selected such that every household had an equal probability of being included in the survey. Net use was determined by asking which individuals slept under a net during the night prior to the survey. Socioeconomic status was assessed using an index of household ownership of assets and housing conditions, and households were divided into five equal-sized groups ("quintiles") according to their index value. A full description of the methods for the socioeconomic evaluation has been published [19]. The data for this study were analysed using STATA 9.0 software.

### Net coverage level within households

Net insecticide treatment status was classified as follows: any net (untreated or treated nets), untreated, expired treatment (net that was treated 12 or more months before use), and ITN (net that was treated less than 12 months before use). Household net coverage was classified as complete, partial, or zero, according to whether all, some, or none of the household members slept under a net. This was repeated for both "any nets" and ITNs amongst five levels of socioeconomic status.

Finally, to determine the person-to-net ratio necessary to obtain complete coverage by nets within net-owning households with at least one infant or young child under age five, the percentage of people sleeping under a net was calculated together with the person-to-net ratio (number of people/number of nets) in that household.

### Net characteristics by person-type

In order to determine the vertical equity of net distribution within households, individuals were divided into seven categories of "person-type" by gender and age: infants (<1 year), young children (1-4 years), older children (5-14 years), adult males (≥15 years), adult non-pregnant females (15-49 years), adult pregnant females (15-49 years), and older females (≥50 years).

These person-types were cross-tabulated with the following variables which may have influenced who was sleeping under a net: number of net holes, net age, insecticide treatment status, and whether a voucher was used to obtain the net. The relationship between person-type and net treatment status was also assessed specifically in households with at least one untreated net, at least one ITN, and at least one infant or young child under five years old.

In the survey, holes were classified into head-sized holes, hand-sized holes, and finger-sized holes, and then counted. To aggregate these into an overall hole index, we used the ratio between the observed numbers of holes of different sizes (relative to the number of finger-sized holes), on the assumption that this was a reasonable proxy for the relative rate at which the different classes of holes accumulate in domestic use. Among those nets in which a complete count of holes was made, there were 3.0 times as many finger-sized holes as hand-sized holes, and 12.9 times as many finger-sized holes as head-sized holes. The hole index for each net was calculated as (number of finger-sized holes + 3 × number of hand-sized holes + 12.9 × number of head-sized holes). Nets that were recorded as having "more than ten" holes were assumed to have 11 holes. Nets that were recorded as having "too many holes to count" were assumed to have an index of 44 finger-sized holes, which was the average number of holes per net in the top 10% of nets when the nets with complete counts were ranked by their hole index.

Additionally, a net age recorded in the survey as "greater than three years" was conservatively assigned a value of 3.5 years.

### Multivariate analysis: relationship between hole index per bed net and person-type

A multivariate analysis was also undertaken to assess vertical equity in the use of nets in good physical condition within households. The dependent variable was the calibrated number of holes per net as calculated by the hole index. The main independent variable of interest was person-type. Variables that could be possible confounders for the relationship between person-type and hole index were as follows: net age, treatment status (never treated, expired treatment, ITN), net size (3.5 ft × 6 ft, 4 ft × 6 ft, 6 ft × 6 ft), number of sleepers under the same net, number of birds (chickens or ducks) owned by the household, roof type (iron sheets or tiles; thatch, grass, or leaves; other), net source, whether or not a voucher was used, the socioeconomic status, the person-to-net ratio in the household, and the type of residence (rural, semi-urban, urban). Domestic birds were included because they sometimes search for fallen insects inside houses and may damage nets; rodents were included because they are known to bite out pieces of netting for nesting material.

The relationship between these potential confounders with the hole index was first assessed by calculating the mean hole index per net and testing for difference in means among the categories of each potential confounder. Next the hole index was categorized into the following: 0 holes, 1-3 holes, 4-8 holes, 9-25 holes, and more than 26 holes. This measure of the hole index by categories was cross-tabulated with all potential confounders, and a Chi-squared test for heterogeneity was used to determine which variables were associated with the hole index. Person-type was then cross-tabulated with the potential confounders while performing a Chi-squared test to determine which variables were associated with person-type. Variables that showed evidence of association with the hole index and person-type were deduced to be confounders and retained in the final model.

We used a negative binomial model for the multivariate analysis because of evidence of overdispersion of the hole index (the variance exceeded the mean). The strength of confounders identified in the bivariate analysis was reconfirmed, and a Poisson regression model was also fitted for comparison (results not shown).

## Results

### Net coverage level within households

The survey covered 6,260 households containing 30,273 people and 6,939 nets. The percentage of households with zero, partial, and complete coverage by any net was determined. In 58% of households, all household members did not use any net (zero coverage). Only 22% (95%c.i. 19,22) of households were completely covered by nets of one kind or another; while a similar number of households were partially covered (21%; 95%c.i. 21,22). In an even larger proportion of households (78%), no one was using an ITN (zero ITN coverage). A smaller number of households were found to be partially covered by ITNs (13%; 95%c.i. 12,14), and an even lower number were completely covered (9%; 95%c.i. 7,10).

The poorest households had much lower coverage, compared with the least poor, in terms of both "partial coverage" and "complete coverage." For "any nets," 8% of the poorest quintile had complete coverage compared with 46% of the least poor. For ITNs, 2% of the poorest quintile had complete coverage compared with 20% of the least poor.

**Figure 1:**
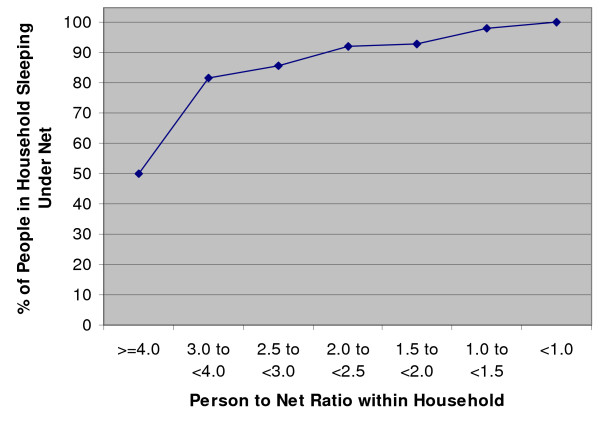
**Percentage of people who slept under any net in relation to person-to-net ratio within households **Among net-owning households with at least one infant or young child under five years old

By calculating the percentage of people sleeping under any net in relation to the household person-to-net ratio within net-owning households with at least one infant or young child under age five, an estimate of the person-to-net ratio needed to ensure complete coverage (every person sleeps under a net) was determined. At least 90% coverage was seen in households with less than 2.5 people per net, while at least 80% coverage was seen in households with less than four people per net (Figure 1). However, in households with four or more people per net, only 50% of household members were using a net.

### Net characteristics by person-type

**Figure 2:**
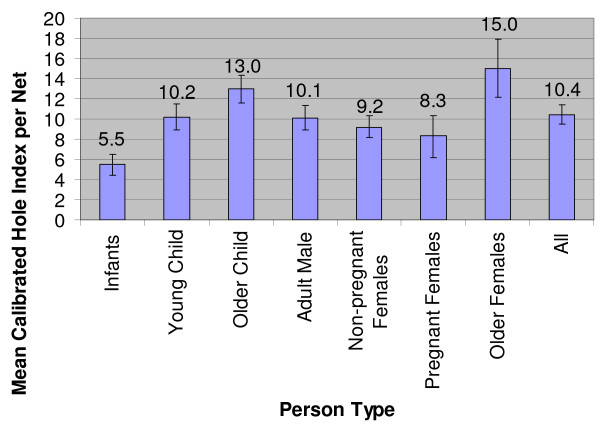
Mean calibrated hole index value per bed net by person-type

Among all those who slept under any net, 42% of individuals slept under nets with no holes. Infants were most likely to sleep under an intact net (54%) in comparison to other person-types. On average, infants slept under nets with the fewest holes (mean hole index = 5.5 holes per net); while older children (mean hole index = 13.0 holes per net) and older females (mean hole index = 15.0 holes per net) slept under nets with the most holes. Young children (mean hole index = 10.2 holes per net) and all older person-types slept under nets with more holes than infants (Figure 2).

**Figure 3:**
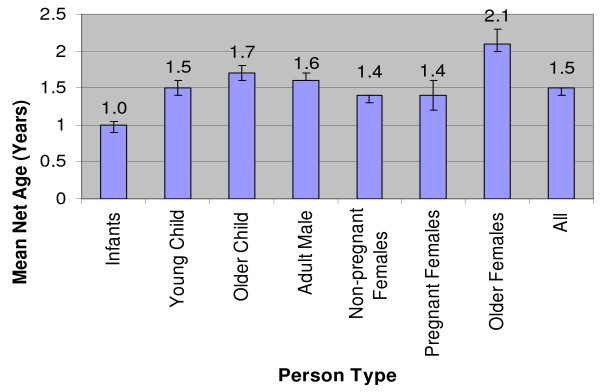
Mean net age (years) by person-type

Infants slept under the newest nets (mean 1.0 year); while older children (mean 1.7 years) and older females (mean 2.1 years) used the oldest nets (Figure 3). Of those that slept under a net, the target (vulnerable to malaria) groups slept under the newest nets with 72% of infants, 57% of currently pregnant females, and 54% of young children sleeping under a net less than 12 months old.

**Figure 4:**
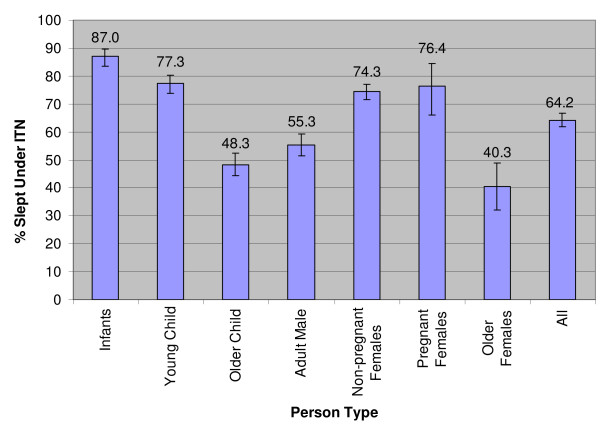
**Percentage of people that slept under ITN by person-type**. Within households with at least one untreated net, at least one ITN, and at least one infant or young child under five years old

Among households with at least one untreated net, at least one ITN, and at least one infant or young child, the evidence suggests that infants (87%) and young children (77%) were most likely to be using an ITN; whereas older females (40%) and older children (48%) were least likely to be using an ITN (Figure 4). Likewise, within these households, older females (42%) and older children (40%) were most likely not to sleep under a net at all (Table 1).

**Table 1:** Net Treatment Type by Person-type, Row%, p < 0.0001, [95% CI]

Person-type	Did not use a net	Never-treated net	Expired Treatment	Insecticide-treated net (ITN)	Total Number
**Infants (<1 y)**	6.9 [4.9,9.7]	3.9 [2.4,6.5]	2.2 [1.2,4.2]	87.0 [83.5,89.7]	406

**Young Children (1-4 y)**	12.88 [10.6,15.6]	6.1 [4.5,8.3]	3.8 [2.8,5.1]	77.3 [73.9,80.3]	1064

**Older Children (5-14 y)**	40.0 [35.6,44.6]	6.1 [4.4,8.6]	5.5 [4.1,7.5]	48.3 [44.2,52.4]	1480

**Adult Males (≥15 y)**	35.7 [31.5,40.1]	6.2 [4.6,8.3]	2.8 [1.9,4.2]	55.3 [51.4,59.2]	1032

**Non-Pregnant Females (15-49 y)**	17.4 [14.7,20.3]	4.9 [3.6,6.6]	3.4 [2.5,4.6]	74.3 [71.6,76.9]	1204

**Pregnant Females**	14.6 [8.9,23.1]	3.4 [1.1,10.0]	5.6 [2.5,12.3]	76.4 [66.0,84.4]	89

**Older Females (≥50 y)**	42.2 [34.0,50.9]	11.0 [6.9,17.3]	6.5 [3.7,11.2]	40.3 [32.2,48.9]	154

**TOTAL**	1412 26.0% [23.4,28.8]	315 5.8% [4.7,7.2]	216 4.0% [3.2,4.9]	3486 64.2% [61.8,66.6]	5429

Within households with at least one untreated net, at least one ITN, and at least one infant or young child under five years old

Infants were the most likely person-type to use a net purchased using a TNVS voucher (49%). Among nets that were obtained by a TNVS voucher, the majority of these nets were shared by a mix of target and non-target groups (86%). Only 12% of nets purchased by vouchers were used exclusively by non-target groups.

### Multivariate analysis: relationship between hole index per bed net and person-type

Net age, net size, number of sleepers under the same net, treatment status, net source, whether the net was purchased using a voucher, and socioeconomic status of the household were strongly associated with both the number of holes per net and person-type (p < 0.05). This association was reconfirmed by fitting each individual confounder and the hole index into a negative binomial regression while adjusting for person-type.

Before adjusting for confounders, all incidence rate ratios for each person-type were greater than 1, suggesting all person-types slept under a net with a greater hole index value than infants. Because of overlapping confidence intervals, the evidence does not suggest a significant difference in hole index value between the person-type categories other than infants.

After controlling for all confounders, the multivariate regression analysis most strongly suggests there were more holes per net in nets used by young and older children in comparison to nets used by infants. For young children, the mean hole index value was 1.3 times greater (95%c.i. 1.0,1.6; p = 0.031) in comparison to nets used by infants. Similarly, older children slept under a bed net with 1.3 times the hole index value (95%c.i. 1.1,1.6; p = 0.01) compared to infants. The data weakly suggest the hole index value was 1.3 times more per bed net (95%c.i. 0.99,1.8; p = 0.055) for older women. The data also weakly indicate the hole index value was 1.2 times more per bed net for adult males (95%c.i. 0.98,1.5; p = 0.074) and non-pregnant females (95%c.i. 0.98,1.5; p = 0.071). However, there was no evidence that there was a greater hole index value per net (p = 0.481) for nets used by pregnant women compared with nets used by infants (Table 2).

**Table 2:** Multivariate analysis: hole index value per net by person-type adjusted for confounders

Hole index	Coefficient	Coefficient 95% CI	Incident Rate Ratio (e^coefficient)	Incident Rate Ratio 95% CI	P value
**Overall**	-------	-------	-------	-------	**F = 22.63 p < 0.0001 α = 3.2 (2.9,3.6)**

**Constant**	**1.7**	[1.2, 2.2]	-------	-------	**<0.0001**

**Person Type**					

**Infants (<1 y)**	-------	-------	-------	-------	-------

**Young Children (1-4 y)**	**0.25**	[0.024,0.48]	**1.3**	[1.0,1.6]	**0.031**

**Older Children (5-14 y)**	**0.28**	[0.070,0.50]	**1.3**	[1.1,1.6]	**0.01**

**Adult Males (≥15 y)**	**0.19**	[-0.018,0.39]	**1.2**	[0.98,1.5]	**0.074**

**Non-Pregnant Females (15-49 y)**	**0.19**	[-0.016,0.39]	**1.2**	[0.98,1.5]	**0.071**

**Pregnant Females**	**0.18**	[-0.31,0.66]	**1.2**	[0.73,1.9]	**0.481**

**Older Females (≥50 y)**	**0.28**	[-0.006,0.57]	**1.3**	[0.99,1.8]	**0.055**

**Used Voucher**	**-0.79**	[-1.11,-0.47]	**0.45**	[0.33,0.63]	**<0.0001**

**Net Age**	**0.042**	[0.035,0.049]	**1.04**	[1.04,1.05]	**<0.0001**

**Net Size**					

**3.5 ft × 6 ft**	-------	-------	-------	-------	-------

**4 ft × 6 ft**	**-0.28**	[-0.50,-0.062]	**0.76**	[0.61,0.94]	**0.012**

**6 ft × 6 ft**	**-0.32**	[-0.57,-0.066]	**0.73**	0.57,0.94]	**0.014**

**Net Source**					

**Shop**	-------	-------	-------	-------	-------

**Hawker/Shifting**	**0.14**	[-0.074,0.35]	**1.1**	[0.93,1.4]	**0.204**

**Health Facility/Gov/NGO**	**-0.22**	[-0.50,0.055]	**0.80**	[0.61,1.1]	**0.115**

**Gift/Other/Not Sure**	**-0.22**	[-0.55,0.10]	**0.80**	[0.58,1.1]	**0.172**

**Treatment Status**					

**Never Treated**	-------	-------	-------	-------	-------

**Expired Treatment**	**0.10**	[-0.14,0.34]	**1.1**	[0.87,1.4]	**0.407**

**ITN**	**0.09**	[-0.10,0.29]	**1.1**	[0.90,1.3]	**0.345**

**# of Sleepers Under Same Net**	**-0.067**	[-0.16,0.022]	**0.93**	[0.85,1.0]	**0.14**

**Socioeconomic Status**					

**SES Quintile 0 (poorest)**	-------	-------	-------	-------	-------

**SES Quintile 1**	**-0.23**	[-0.60,0.15]	**0.80**	[0.55,1.2]	**0.237**

**SES Quintile 2**	**0.016**	[-0.38,0.41]	**1.0**	[0.68,1.5]	**0.935**

**SES Quintile 3**	**-0.17**	[-0.54,0.20]	**0.85**	[0.59,1.2]	**0.378**

**SES Quintile 4 (least poor)**	**-0.31**	[-0.70,0.074]	**0.73**	[0.50,1.1]	**0.112**

Adjusted for the following confounders: whether the net was purchased using a voucher, net age, net size, net source, treatment status, number of sleepers under same net, and socioeconomic status of the household

## Discussion

### Diversion effect

With 21% of households partially covered by any nets and 13% partially covered by ITNs, there was a percentage of households partially covered by untreated or expired treatment nets, potentially placing individuals in these households not sleeping under a net at higher risk due to the mosquito "diversion effect" [12]. Conversely, in the households partially covered by ITNs, the insecticide treatment is expected to give protection not only to those sleeping underneath the nets but also to non-net-users within the same households. Therefore, individuals not sleeping under a net may still have had some protection from nearby ITNs. Additionally, even if the net has holes, treating the net with insecticide will provide some protection for the user against mosquitoes [21].

These results underline the need for greater coverage of long-lasting insecticidal net (LLIN) products or greater treatment of conventional polyester nets with longer-lasting insecticide (insecticide plus binder). If more nets are treated and remain effective for a longer period of time, risks associated with the "diversion effect" could be decreased across all socioeconomic groups, especially among the poorest households who are least likely to use ITNs. The newly introduced LLIN voucher should help to increase the share of treated net use.

Among net-owning households with at least one infant or young child under age five, at least 80% coverage was achieved in households with at least one net for every four people. Because of the tendency for household members to share a net and the additional protection provided by ITNs to non-net-users in the same household, determining the specific person-to-net ratio within households could be useful in assessing household coverage by nets or ITNs as more distribution systems aim to achieve universal coverage.

### Vertical equity

Because infants were most likely to use new, intact ITNs, vertical equity, in which household members at highest risk receive the best protection, was observed for infants. This finding is similar to that seen in Netmark surveys among households with at least one net and one child under age five, which also found that infants used nets more than all other person-types in Nigeria, Zambia, Mozambique, and Mali [22-25]. These studies provide further evidence that infants are prioritised for net use over non-target groups such as adult males, and vertical equity for infants was achieved within households. Furthermore, the TNVS is shown to be successful in reaching the target population as only 12.1% of TNVS nets were used exclusively by non-targeted individuals. Infants will have further benefited with the expansion of the TNVS in 2007 to include an infant voucher given to the caretaker when nine-month-old infants are brought to reproductive and child health clinics (RCH) for measles vaccination [26].

However, more worrying is the use of nets with more holes by young children, who are also at considerable malaria risk. With adjustment for all confounders, the evidence suggested young and older children were sleeping under nets with more holes than infants, which is consistent with the relative vulnerability of these age groups to malaria. Among older person-types, there were no significant differences between the mean hole indices.

In households with at least one untreated net, at least one ITN, and at least one infant or young child, the probability of using an ITN was highest among infants, followed by young children and women of reproductive age, then adult males and older children, and least among older women. In other words, the rank order of priority for ITN use among person-types broadly matched their relative vulnerability to malaria.

Older children were using nets in worse-than-average condition in terms of number of holes and age and were less likely to use a net or ITN in households with at least one untreated net, at least one ITN, and at least one infant or young child. This decreased likelihood for older children to use protective nets may be a result of insufficient nets within the household.

### Net condition and protection

Whether treated or untreated, intact nets still offer some protective benefit against mosquitoes in comparison to no nets at all [27]. In a previous study in Tanzania, an "intact net" was defined as less than 20 holes that are less than 2 cm in diameter [28]. By this definition, 83% of nets in the present study were "intact," assuming that finger-sized holes were less than 2 cm in diameter. Therefore, most nets in this study were giving some degree of protection against mosquito bites and risk of malaria.

Nevertheless, better protection is provided to the user when there are fewer holes in the net. ITN programmes in Tanzania such as the TNVS have succeeded in covering infants, who were most likely to sleep under an intact net. Young children are expected to benefit from the free "catch-up" net campaign, which started in 2008, targeting all children under five years old.

The data suggest that the TNVS malaria control programme has been effective in reaching infants by targeting pregnant women. Recent programme developments, including the completion of a national distribution of free LLINs to all children under five will have gone some way towards raising ITN coverage in this group. A universal coverage campaign, targeting all "sleeping places" in every household in the country with LLINs, will begin in late 2010. In order to encourage a further increase and improvement in the use of ITNs, information, education, and communication (IEC) campaigns should also address the issue of increasing vertical equity of net use within households by net condition and treatment status.

However, using an IEC campaign alone or making LLINs more affordable is not sufficient to change human behaviour. It is also important to understand the context behind decision-making within households [29]. For example, in a study to determine willingness to pay for hypothetical malaria vaccines in Burkina Faso, the community had a stronger preference for a vaccine protecting pregnant women than young children due to the perception of the greater importance of women in the livelihood of the household [30]. Further studies would be necessary to explore preferences for net assignment based on condition and treatment status. In-depth interviews and focus groups would lead to a greater understanding of the motivations and constraints of behaviour within households to determine within-household preferences for appropriate malaria prevention methods. This research could reveal determinants of the disparities observed in net use between children and infants in order to identify specific targets to resolve this problem. For example, one testable hypothesis is that children aged one to four years are given priority while they remain the youngest child, but cede this priority to the new baby if and when a younger sibling arrives in the family.

## Conclusions

Two main findings emerge from this analysis of the 2006 TNVS survey data. First, small decreases in the household person-to-net ratio resulted in proportionately large increases in within-household net coverage levels, reducing chances for the "diversion effect." Greater than 80% coverage of individuals within households was achieved with a person-to-net ratio of less than four. Second, infants were more likely to use intact ITNs than any other household member. Both young and older children used nets that had more holes than those used by infants. More generally, this study suggests that the more vulnerable-to-malaria members of the family are given priority for use of the most protective nets in the household, and thus, vertical equity was achieved. However, overall coverage remains unacceptably low, and too many households are forced to make difficult decisions about who should sleep without the protection of an effective net.

In addition to the TNVS, the U5 campaign was launched in 2008 to provide LLINs to all children under age five in Tanzania, which will help to improve overall coverage as well as equity. While it is necessary to increase the number of ITNs per household, it is also important to understand how decisions about who sleeps under which net are made within households. Ensuring that household behaviour supports the same goals as those of malaria control programmes to increase the use of intact ITNs by the most biologically vulnerable will allow malaria control interventions to be more effective in protecting the lives of pregnant women, infants, and young children.

## Competing interests

The authors declare that they have no competing interests.

## Authors' contributions

AT carried out the analysis and interpretation of the data and drafted the paper. KH and JL critically revised the paper. JL contributed his expertise on malaria and insecticide-treated nets and gave guidance for the analysis of the data. KH as principal investigator for the Monitoring and Evaluation of the Tanzania National Voucher Scheme provided the data, the idea for this project as well as advice and support. All authors read and approved the final manuscript for publication.
